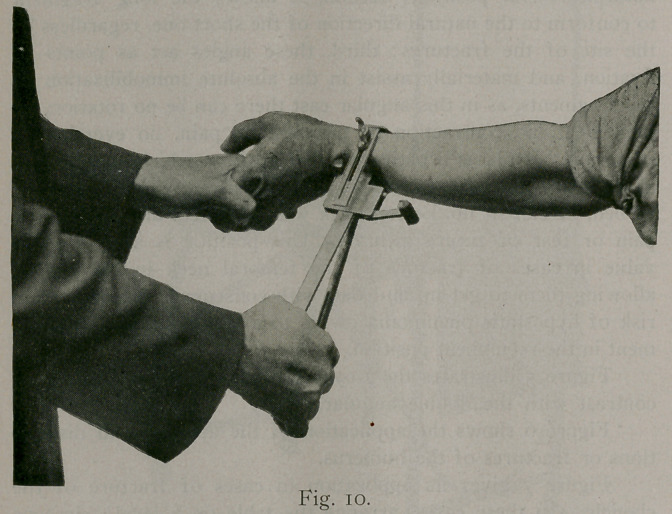# The Perfect Mechanical Reduction of Fractures and Method of Absolute Immobilization

**Published:** 1908-02

**Authors:** J. H. Downey

**Affiliations:** Gainesville, Ga.


### THE PERFECT MECHANICAL REDUCTION OF FRACTURES AND METHOD OF ABSOLUTE IMMOBILIZATION.

BY J. H. DOWNEY, GAINESVILLE, GA.

---

For the last half century the treatment of fractures has utterly failed to keep pace with the other branches of Surgery in its advancement toward the reduction of mortality, relief of human suffering and the lessening of deformities. While a number of Surgeons have spent much time in devising various kinds of apparatus for the retention of the fragment, yet, not until the last few years, has there been any attention paid to the most impirtant part, "Reduction." We have depended wholly on human strength for this purpose and few seem to realize the fact that it was practically a physicial impossibility for man to pull, strong, long and steady enough to reduce the fracture of the lower extremity and apply a proper retention dressing. If anyone wishes to test the truth of this statement, let him take two strong men, hoodwink and put one at each end of a pair of spring household scales that draws 25 pounds, have them pull in opposite direction until the finger points to the 25 Lb. mark,

Fig. 1.

Fig. 2.

and hold it exactly over this point (not beyond). You will find a difference in the reading of from three to ten pounds within twenty or twenty-five minutes. This test will show you conclusively how steady human strength will hold, even under moderate pressure for a length of time, but should this same traction be on a human limb, you would readily see the results.

Fig. 3.

Prior to the discovery of the X ray, if we got within an inch or an inch and a half of its former length, we congratulated ourselves on the good results, the normal tilt of the pelvis fully compensating for the shortening and showed no deformity, and vicious union was thought to be of occasional accident, a few rare specimens of which have been preserved in museums for our study, but since the introduction of this most wonderful light, which renders transparent the tissues overlying the bone, and brings it in perfect view, we are able to study these cases in the living subject, and to our utter astonishment we find a veritable walking museum in the streets of every city throughout the country, showing conclusively, perfect results to be exceedingly rare, and the man, to-day, who is satisfied with the results of his fractures, is one who does not study them under the X ray during repair and after recovery. Prof. Chas. L. Scudder, of Boston, says the object in the treatment of fractures are perfect reduction, perfect immobilization and to resture the limb to its normal usefulness, which is, unquestionably correct. Let us take these two objects, perfect reduction and perfect immobilization, and study them carefully: first, reduction, without this prerequisite, as we all know good results are impossible, yet all authors have very little to say as to how to do it and hold until a perman-

Fig. 4.

ent dressing is applied. They simply tell you how to grasp the limb and to pull until the ends of the fragment are in opposition. Now, if you will read between the lines, they will tell you that you have failed at reduction and you must prepare for mechanical traction by weight or suspension swing to gradually overcome muscular action, and complete the reduction some hours, or days, later, and that this mechanical traction must be kept up for weeks until union is sufficient to prevent over-riding of the fragments. This continuous traction for weeks tells us at once that the method taught for this second object (immobilization) is imperfect, if not imperfect, why should we apply traction to an immoble object, if it was immovable, what good would traction do? If not immovable, what man is there that can tell the amount of weight necessary to exactly overcome muscular action and bring the fragment in oposition, and how shall we tell

Fig. 5.

how to keep it, without varying, for the space of three weeks and how shall this traction be kept up while making the necessary movements to attend the calls of nature, bathing, etc? I must confess these are all questions that look to me beyond human

Fig. 6.

comprehension. It leaves to judge by comparison and the daily measurement and to blindly trust in Providence that all will be well in the end. I have not spoken of the teachings of our text book in a spirit of criticism by any means, but, as they have appeared to me in their clinical application,—very imperfect.

Fig. 7.

Is it possible then, for us to carry out the two great objects of Scudder in the treatment of fractures? Yes! By the proper mechanical reduction at first and then the application of a well fitted dressing to the limb, flexed at such angles as to give normal muscular relaxation and the angle to form point of fixation to prevent over riding of bone. Any dressing that will do this may be used. I, myself, prefer plaster of paris applied by section as described in its application to the following fractures:

First, Pott's fracture. Arrange the table as shown in figure 1. Place the patient on the table, pad the foot to the ankle with cotton wadding and apply a plaster slipper in the usual way. Allow this to set and when sufficiently hard, put on the leather cuff around the ankle, as shown in figure 1, and make your traction until the fragments are in position. Then drop the lower section on the injured side, pad the leg with wadding, and apply plaster of paris in the usual way to the knee.

Second, fractures of the upper third of the tibia and fibula. Arrange the top of the table as in figure 2, with the lateral half dropped and the foot-piece elevated. Put on the plaster slipper, as before, pad the knee with cotton wadding, and apply plaster of paris in an elbow with the knee flexed in the desired position. Place the patient on the table and arrange the traction apparatus in the form of a tripod over the patient, as shown in figure 2. Make traction until the fractured ends are in their position, and fill in the interspace in the usual way. It makes these welding points a little stronger to have strips of copper wire woven in between the layers at this point, giving extra strength with a mini-

Fig. 8.

mum weight. Note here the angle at the knee, as shown by the accompanying cut, and the heel and instep to prevent any shortening.

Figure 3 shows a complete dressing for fractures of the middle and upper third of the tibia and fibula, also the first section in case of a fracture of the femur. In fractures of the femur anywhere from the neck to the condyles, always use the double angular plaster splint, for reasons given hereafter. In this class of fractures, arrange the table as shown in figure 3, with the lateral half on the injured side dropped down on the sliding bar and the lower section of that elevated as a foot rest. Place the patient as in fig. 3, with hips on the oval hip rest pad center post against the perineum; arrange the traction apparatus, as shown in the figure, until the pulley rope makes traction in the axis of the femur. With the first section applied and set (figure

Fig. 9.

3) arrange the leather cuff so as to grasp the condyles of the femur over the plaster, and make traction slowly and steadily until the fractured ends are in position. Then pad the legs, hips, abdomen and lower part of the chest to the transverse nipple line with cotton wadding, telescope the lower half of the upper section, and apply the plaster in the usual way up to the nipple line. After having applied a couple of layers, insert a strip of copper gauze wire, about 6 or 8 inches wide, and long enough to reach from under the thigh up to the top of the cast. After this is fitted, apply another layer of the plaster roller. The copper wire gauze inserted at this point gives extra strength opposite the hip joint where the cast is only three-quarters of a circle and most likely to give way.

When this is complete, you have a dressing as shown in figure 4, the dougle angular plaster splint, which has many points to commend it to the profession: first, it places every muscle in a natural, restful position; second, it allows the long fragment to conform to the natural direction of the short one, regardless of the site of the fractures; third, these angles act as points of fixation, and materially assist in the absolute immobilization of the fragments, as in this angular cast there can be no rotation, no movement, no contraction of muscles, no pain, no eversion or inversion. It places a patient in that position which enables him to make any movement that he may desire. He can get in and out of bed, he can sit up, he can walk with a crutch, and all without pain or fear of future injury. This position is also of great value in cases of fracture of the femoral neck in old people, allowing them to get up and out, with consequent freedom from risk of hypostatic pneumonia owing to a long-continued confinement in the recumbent position.

Figure 5 illustrates the Royal Whitman position, by way of contrast with the double angular.

Figure 6 shows the application of the apparatus in dislocations or fractures of the humerus.

Figure 7 gives its application in cases of fracture of the clavicle. In these cases, arrange the table on a level, telescope the lower half of the upper section, remove the center post, and hook the spinal brace into the place from which the center post was removed. Then place the patient on a table, the spinal brace reaching about the sixth cervical vertebra; put a pillow under the head, and pad the chest with cotton wadding from the nipple line up over the shoulders. Next, tie the sound shoulder to the table in the desired position; then take the injured shoulder and bring it backward, upward, and outward until the fragments are in proper position, and fasten it by means of a roller bandage.

With the patient fixed in this position, it is impossible for the fragments to get out of place. Begin at the nipple line with a plaster roller, and go up well under the axilla, around the base of the neck, behind, in front of the shoulder on the opposite side, around the front of the neck, and back of the shoulder on the opposite side, thus making a double figure of eight back and front or at each shoulder. Then, with a back-and-forth motion from front to back on either side of the neck, fill in the yoke. Apply over this another layer of the roller, and again make a double figure of eight around the neck and shoulders. Thus you have both shoulders in an absolute fixed position, the sound shoulder

Fig. 10.

acting as a fulcrum to keep the injured one on the same level. This is a painless and efficient method of retaining a fractured clavicle, affording better results than any apparatus or dressing I have hitherto known. It is perfectly comfortable and allows a man to dress as usual with his arm in his sleeve, the only necessary outer dressing being a simple sling.

Figure 8 is presented here simply to show that the apparatus can be used in the application of a plaster jacket, which gives the most perfect position, the most thorough access to the patient of anything I have tried.

Figure 9 shows that this table combines all of the advantages described in connection with the previous cuts with a good general operating table with easily adjusted Trendelenburg position stirrup leg rest and for office work or for special operations.

My object being to give the surgeon and young practitioner an operating table with attachments that would render him such assistance in the way of traction and access to his patient, without movement, that it would enable him to give his patient far more comfort, greater freedom and better results than he could possibly do with any number of trained assistants, and all with much less worry to himself, thereby protecting both his reputation and purse.

In this method, I carry out all of the cardinal principles taught in our text books of to-day; but with this perfect method of mechanical reduction, which is otherwise impossible, and the absolute immobilization by well fitted plaster dressing with the limb flexed so as to give muscular relaxation and angles to form points of fixation to prevent the limb telescoping into the cast and consequent over-riding of bone. The difference in comfort, freedom and results are too great to escape the notice of the profession, or even the laity; no surgeon to-day can afford, for many reasons, to put his patient on his back in bed for six weeks and with ten days or two weeks pain, when he can have him sitting and walking about the house in less than six days, free from pain, and in the end get a better result than was possible by any other method. This method should be of unusual interest to the surgeon doing work for corporations, for, by certainty of carrying out the prime object, you lessen deformity and thereby lessen their liability for damage.

Nearly all surgeons say a Colles fracture is one that any one can easily reduce, and yet we see as many bad results from this fracture as from any other and as these cases often come to the surgeon a week or ten days after the injury, it makes it all the more difficult to reduce properly. Seeing a number of these cases which gave me trouble to reduce, I devised a little apparatus for the purpose (Figure 10) which makes even the most difficult case easy and sure of proper reduction.

Fig. 10 shows the apparatus in position on wrist; after it has been adjusted, all that is necessary is to grasp the hand, make quick forcible extension, and press the lever with the other hand, which gives you force sufficient to reduce the most difficult case. It is so quickly done that an anaesthetic is not at all necessary, as the pain is only momentarily; the reduction is perfect and a close fitting plaster of paris or a simple posterior P. P. splint is all that is necessary and your result will be perfect. This is not a necessity, but it makes the cases easy without an anaesthetic, which must be done sometime, owing to being alone, or disease which renders an anaesthetic hazardous, and I merely make mention of it here; others can do as they like. It has certainly saved much time, inconvenience and worry, and my patient suffers little pain and gets perfect results and have used no anaesthetic.

## Figures and Tables

**Fig. 1. f1:**
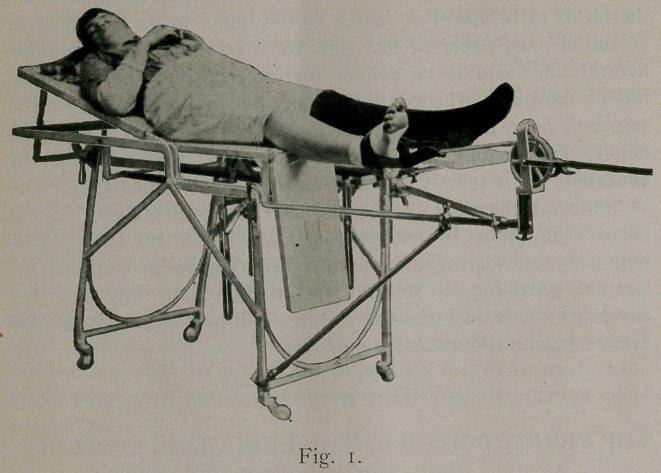


**Fig. 2. f2:**
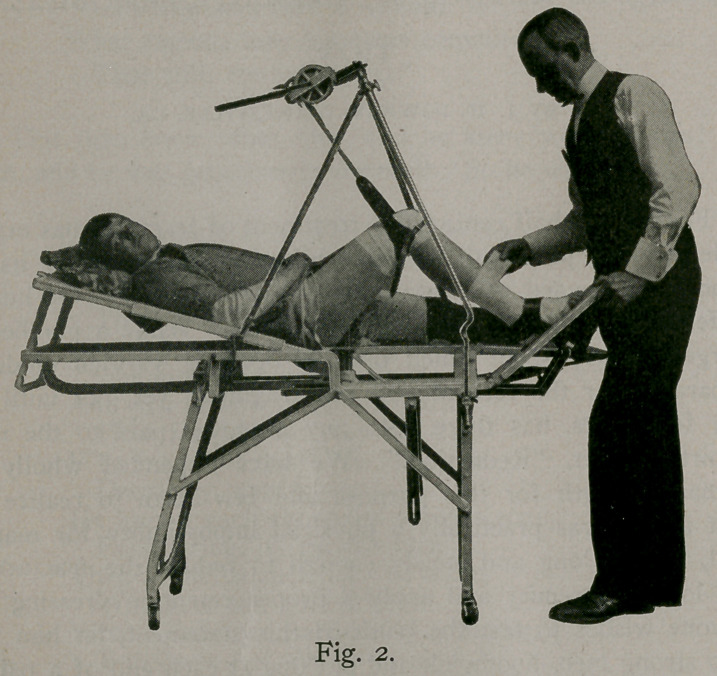


**Fig. 3. f3:**
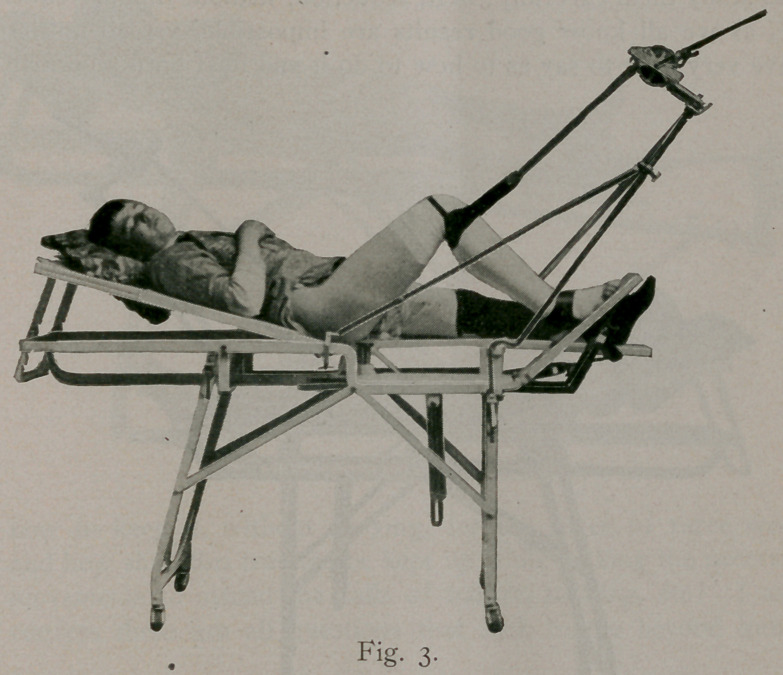


**Fig. 4. f4:**
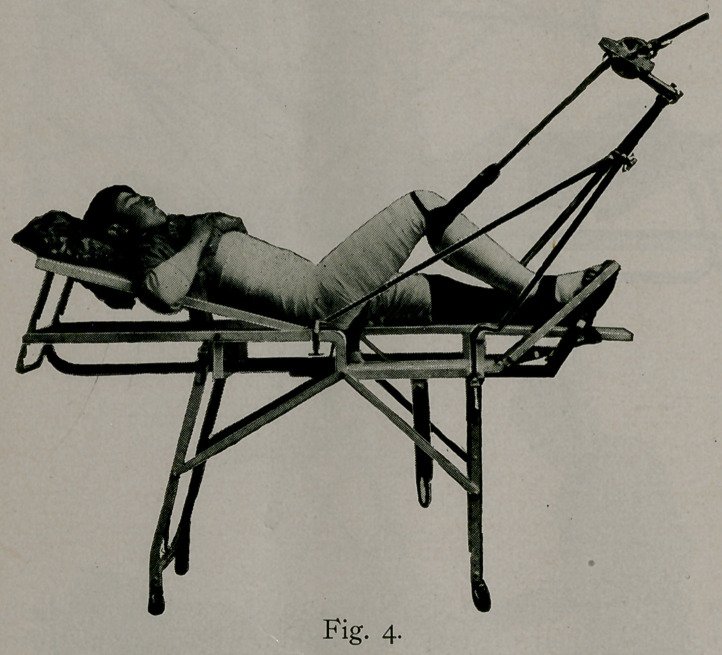


**Fig. 5. f5:**
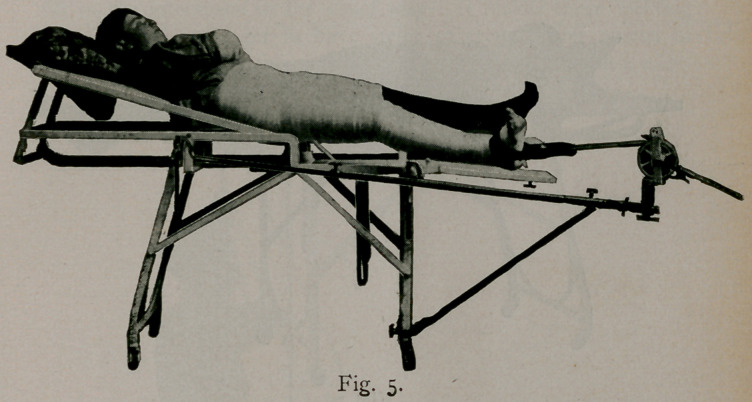


**Fig. 6. f6:**
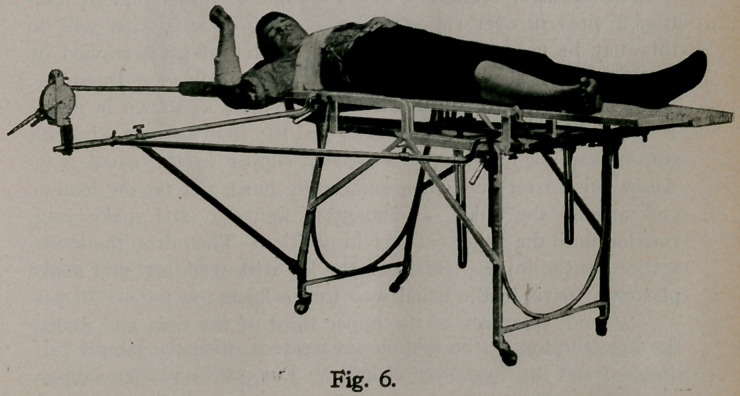


**Fig. 7. f7:**
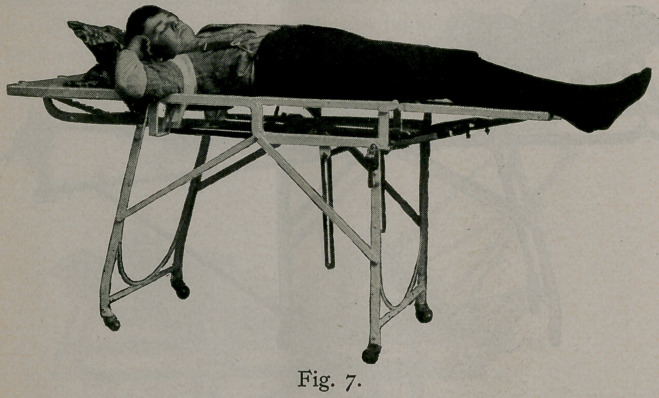


**Fig. 8. f8:**
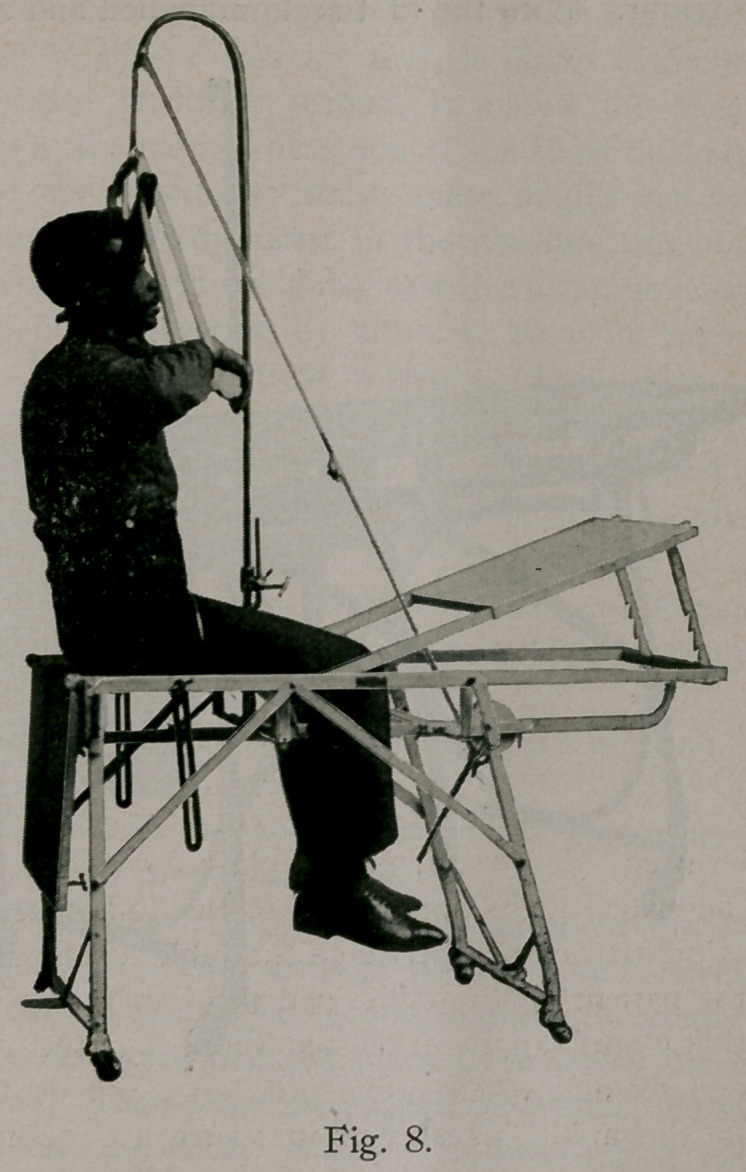


**Fig. 9. f9:**
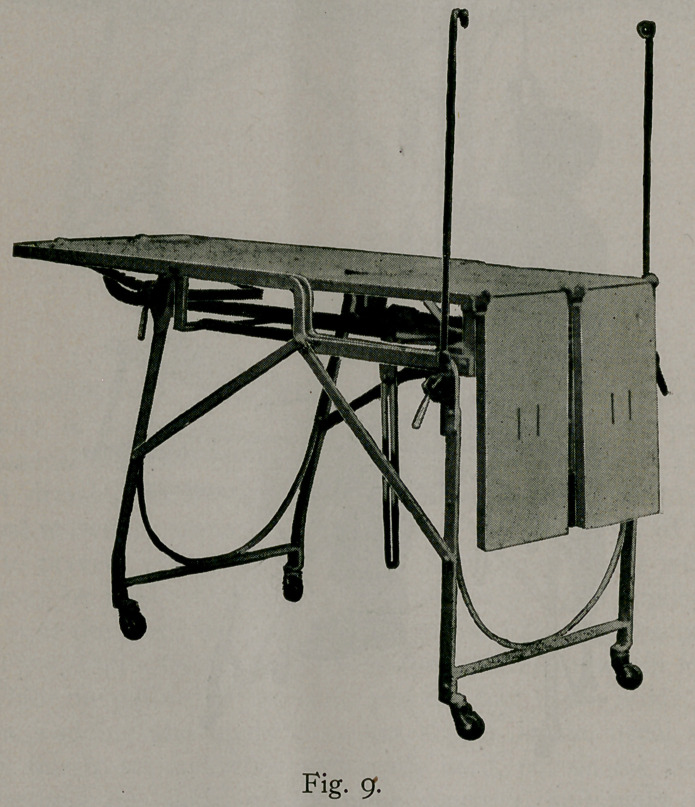


**Fig. 10. f10:**